# Supplementary material for: Basolateral and central amygdala orchestrate how we learn whom to trust
Source: Commun Biol. 2021 Nov 25;4:1329. doi: 10.1038/s42003-021-02815-6 (PMC8617284; doi:10.1038/s42003-021-02815-6)
Supplement: Supplementary file 2 — Supplementary Information [file 42003_2021_2815_MOESM2_ESM.pdf]

Supplementary Information

Validation of functional MRI Data quality

Visual inspection of the EPI images demonstrates sufficient data quality to extract time series from the volumes of interest.

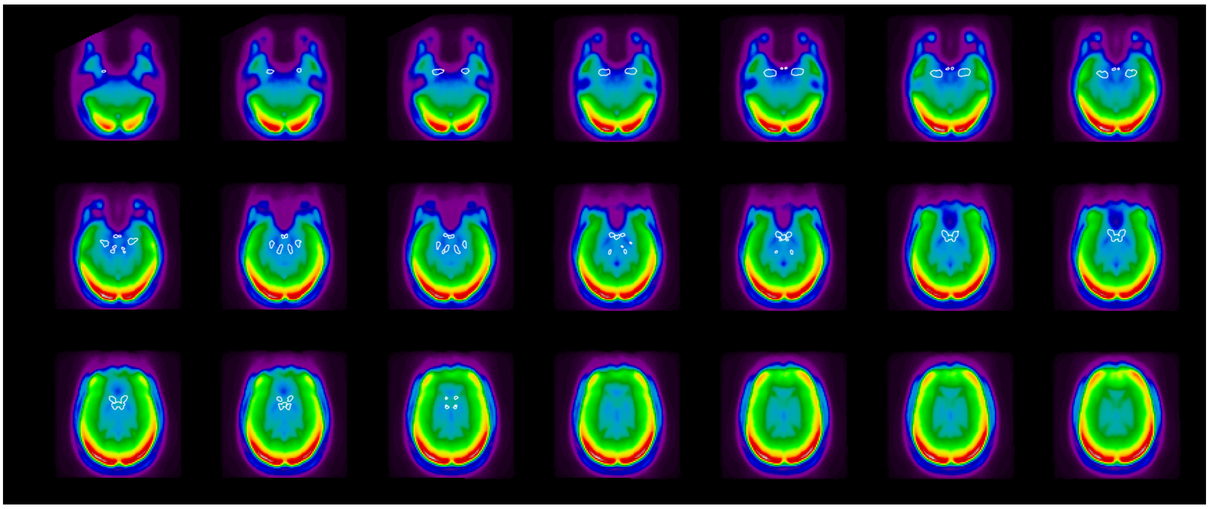

Supplementary Figure 1. Median image of single-subject mean EPI volumes in study space with overlay of the VOI masks (BLA, CeA, NAc, SN/VTA, BST, Basal Forebrain). Instead of grayscale, Matplotlib's *nipy_spectral* colormap is used for better visualization of the signal intensities.

BLA and CeA functional connectivity analysis. Differences in functional connectivity when using the BLA and CeA as seed regions indicate (a) differences in functional brain network and (b) sufficient specificity in the fMRI data to robustly detect functional differences in these subnuclei.

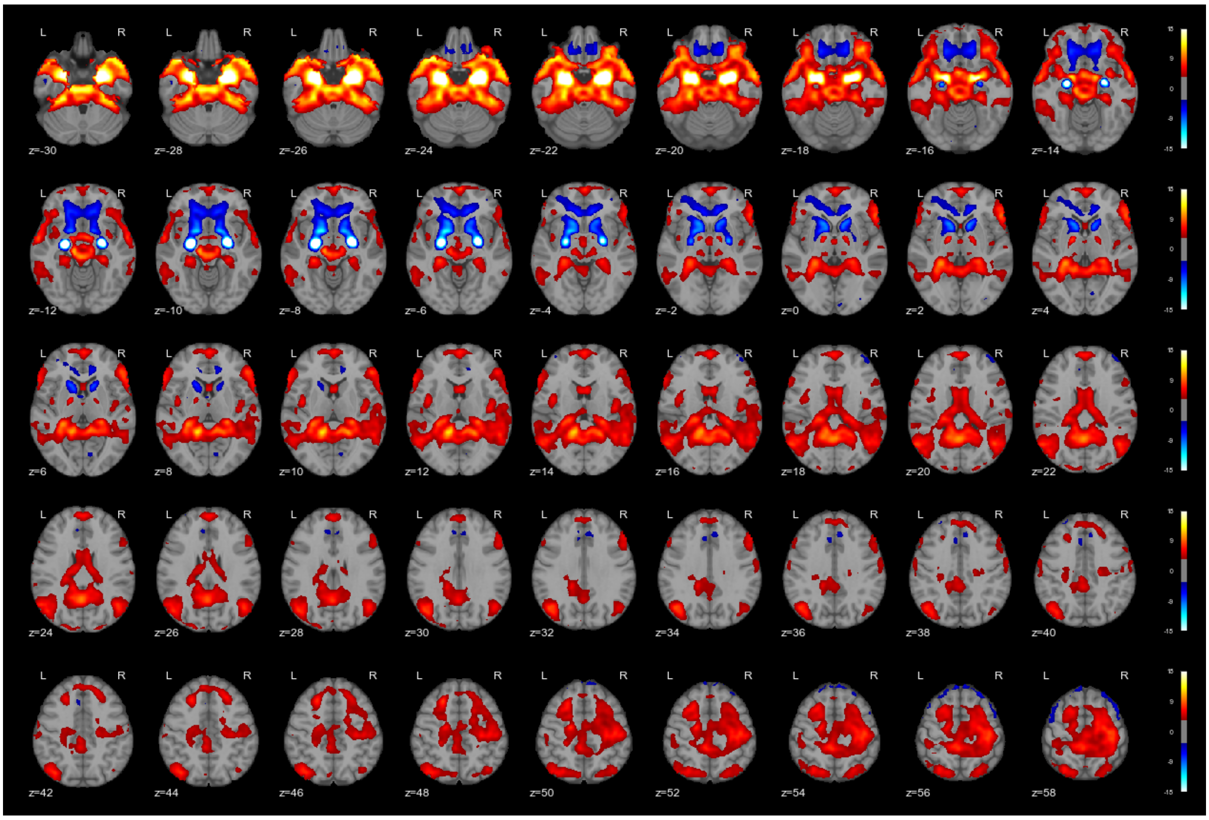

Supplementary Figure 2. Differences in functional connectivity of BLA>CeA (hot) and CeA>BLA (cool).

Investigation of potential habituation effects in the amygdala over rounds

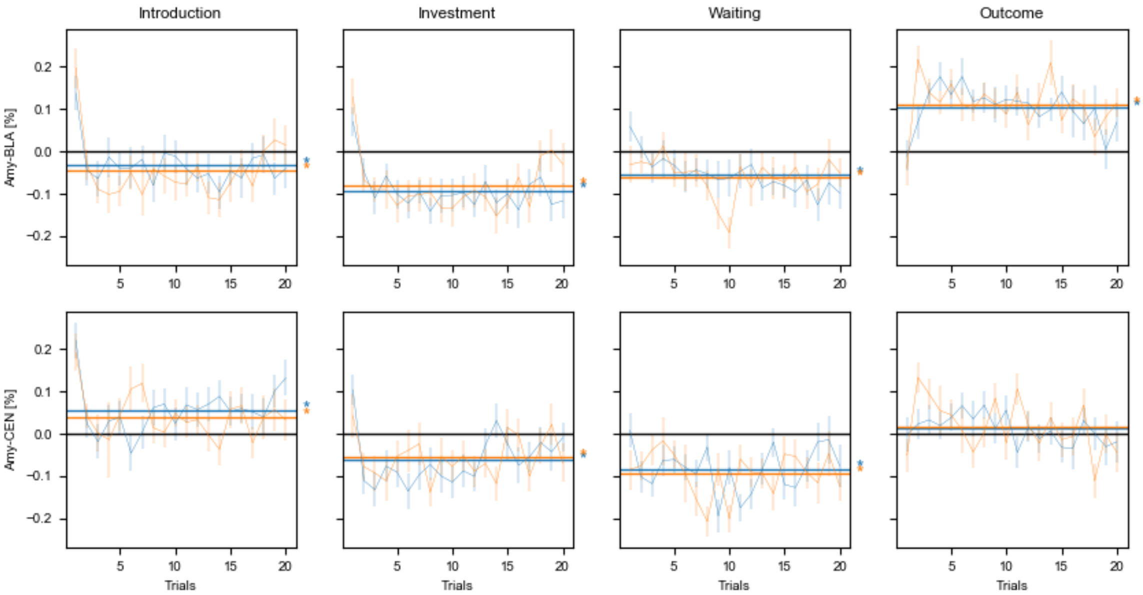

Supplementary Figure 3. No evidence for amygdala habituation. Averaged percent signal change for the different task phases for the trustworthy (blue) and untrustworthy player (orange).

Whole brain analysis for individual task phases

Second-level whole brain analysis of single-subject result was performed using a GLM that included the following conditions: *introduction, investment, waiting, outcome*. In this analysis we did not differentiate between the trustworthy vs. untrustworthy player and learners vs. non-learners.

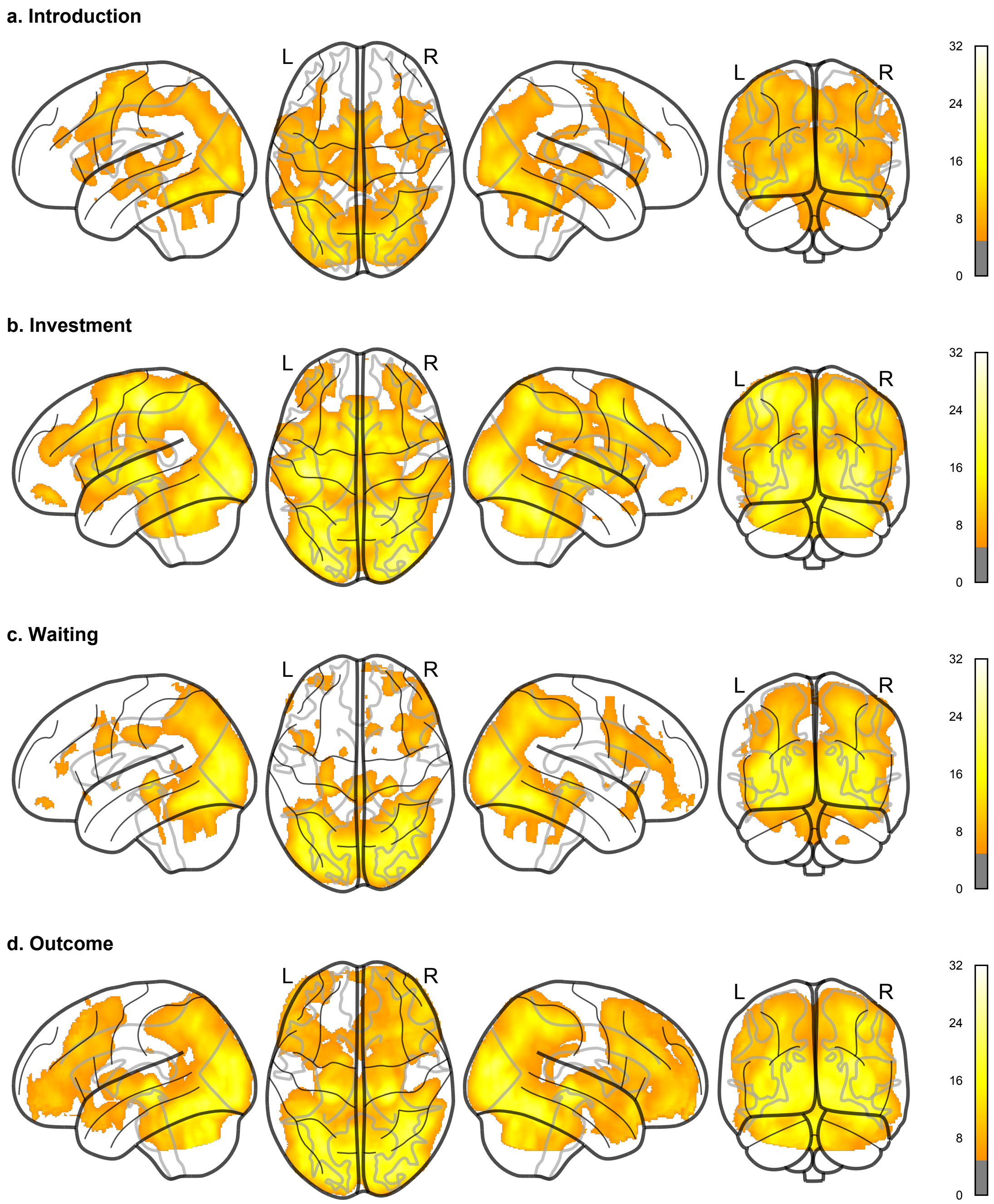

Supplementary Figure 4. SPM of the group-level whole brain results for the *introduction*, *investment*, *waiting*, and *outcome* phases (rows 1 to 4). Threshold was set to $p<0.05$ FWE-corrected (voxel-wise, whole-brain).
