# Supplementary material for: Basolateral and central amygdala orchestrate how we learn whom to trust
Source: Commun Biol. 2021 Nov 25;4:1329. doi: 10.1038/s42003-021-02815-6 (PMC8617284; doi:10.1038/s42003-021-02815-6)
Supplement: Supplementary file 3 — Reporting Summary [file 42003_2021_2815_MOESM3_ESM.pdf]

nature research

Corresponding author(s): Ronald Sladky and Claus Lamm

Last updated by author(s): Aug 31, 2021

# Reporting Summary

Nature Research wishes to improve the reproducibility of the work that we publish. This form provides structure for consistency and transparency in reporting. For further information on Nature Research policies, see our Editorial Policies and the Editorial Policy Checklist.

## Statistics

For all statistical analyses, confirm that the following items are present in the figure legend, table legend, main text, or Methods section.

| n/a | Confirmed | |
|---|---|---|
| ☐ | ☒ | The exact sample size (*n*) for each experimental group/condition, given as a discrete number and unit of measurement |
| ☐ | ☒ | A statement on whether measurements were taken from distinct samples or whether the same sample was measured repeatedly |
| ☐ | ☒ | The statistical test(s) used AND whether they are one- or two-sided<br>*Only common tests should be described solely by name; describe more complex techniques in the Methods section.* |
| ☐ | ☒ | A description of all covariates tested |
| ☐ | ☒ | A description of any assumptions or corrections, such as tests of normality and adjustment for multiple comparisons |
| ☐ | ☒ | A full description of the statistical parameters including central tendency (e.g. means) or other basic estimates (e.g. regression coefficient) AND variation (e.g. standard deviation) or associated estimates of uncertainty (e.g. confidence intervals) |
| ☐ | ☒ | For null hypothesis testing, the test statistic (e.g. *F*, *t*, *r*) with confidence intervals, effect sizes, degrees of freedom and *P* value noted<br>*Give P values as exact values whenever suitable.* |
| ☒ | ☐ | For Bayesian analysis, information on the choice of priors and Markov chain Monte Carlo settings |
| ☒ | ☐ | For hierarchical and complex designs, identification of the appropriate level for tests and full reporting of outcomes |
| ☐ | ☒ | Estimates of effect sizes (e.g. Cohen's *d*, Pearson's *r*), indicating how they were calculated |

*Our web collection on statistics for biologists contains articles on many of the points above.*

## Software and code

Policy information about availability of computer code

| | |
|---|---|
| Data collection | z-Tree (version 3.3.7) for behavioral data; Siemens Skyra 3 Tesla MRI scanner (Siemens Healthineers, Erlangen, Germany) for imaging data. |
| Data analysis | MATLAB (SPM12), Python (nipype, nilearn, numpy, scipy, matplotlib) |

For manuscripts utilizing custom algorithms or software that are central to the research but not yet described in published literature, software must be made available to editors and reviewers. We strongly encourage code deposition in a community repository (e.g. GitHub). See the Nature Research guidelines for submitting code & software for further information.

## Data

Policy information about availability of data

All manuscripts must include a data availability statement. This statement should provide the following information, where applicable:

- Accession codes, unique identifiers, or web links for publicly available datasets
- A list of figures that have associated raw data
- A description of any restrictions on data availability

The code and all data needed to reproduce the results and figures are published on our lab's public github page (https://github.com/scanunit).

# Field-specific reporting

# Life sciences study design

All studies must disclose on these points even when the disclosure is negative.

| | |
|---|---|
| Sample size | 62 healthy, neurotypical volunteers (age=23.83±3.15 years, f/m=31/31) |
| Data exclusions | none |
| Replication | n/a |
| Randomization | No group allocation, experimental trials were randomized in z-tree |
| Blinding | Not relevant because all participants were part of the experimental group. |

# Reporting for specific materials, systems and methods

We require information from authors about some types of materials, experimental systems and methods used in many studies. Here, indicate whether each material, system or method listed is relevant to your study. If you are not sure if a list item applies to your research, read the appropriate section before selecting a response.

## Materials & experimental systems

| n/a | Involved in the study | |
|---|---|---|
| ☒ | ☐ | Antibodies |
| ☒ | ☐ | Eukaryotic cell lines |
| ☒ | ☐ | Palaeontology and archaeology |
| ☒ | ☐ | Animals and other organisms |
| ☒ | ☐ | Human research participants |
| ☒ | ☐ | Clinical data |
| ☒ | ☐ | Dual use research of concern |

## Methods

| n/a | Involved in the study | |
|---|---|---|
| ☒ | ☐ | ChIP-seq |
| ☒ | ☐ | Flow cytometry |
| ☐ | ☒ | MRI-based neuroimaging |

# Magnetic resonance imaging

## Experimental design

| | |
|---|---|
| Design type | event-related, task-based design |
| Design specifications | 1 session, 1 run, 20 rounds/trials times 2 conditions |
| Behavioral performance measures | investments per trial and subjective rating after experiment |

## Acquisition

| | |
|---|---|
| Imaging type(s) | functional |
| Field strength | 3 Tesla |
| Sequence & imaging parameters | GE, EPI, MB-EPI factor=4, TR/TE = 704/34 ms, voxel size 2.2×2.2×3.5 mm^3, matrix size 96×92×32 voxels, flip angle=50° |
| Area of acquisition | whole brain |

Diffusion MRI ☐ Used ☒ Not used

## Preprocessing

| | |
|---|---|
| Preprocessing software | SPM (SPM12, http://www.fil.ion.ucl.ac.uk/spm/software/spm12/) and the Python projects nipype (http://nipy.org/nipype) and nilearn (http://nilearn.github.io) |
| Normalization | non-linear normalization of the EPI images to a study-specific group template using ANTs |
| Normalization template | first to study-specific in-house template, then to ICBM152 |
| Noise and artifact removal | realignment parameters in GLM; additionally, white matter and CSF signal in the functional connectivity analysis |
| Volume censoring | none |

## Statistical modeling & inference

| | |
|---|---|
| Model type and settings | mass univariate RFX |
| Effect(s) tested | phase-dependent BOLD percent signal change differences between the trustworthy and untrustworthy players - and between learners and nonlearners. |

Specify type of analysis: ☐ Whole brain ☒ ROI-based ☐ Both

| | |
|---|---|
| Anatomical location(s) | BLA and CeA (Tyszka and Pauli, 2016), NAc (AAL Atlas), BST (Torrisi et al., 2015), SN/VTA (Talairach atlas transformed to MNI space), and basal forebrain (Jülich Brain MPM atlas). |
| Statistic type for inference (See Eklund et al. 2016) | Threshold was set to $p<0.05$ FWE-corrected (voxel-wise, whole-brain) for Supplementary Figure 4. |
| Correction | FWE |

## Models & analysis

| n/a | Involved in the study |
|---|---|
| ☐ | ☒ Functional and/or effective connectivity |
| ☒ | ☐ Graph analysis |
| ☒ | ☐ Multivariate modeling or predictive analysis |

| | |
|---|---|
| Functional and/or effective connectivity | Pearson correlation in Supplementary Figure 2. |
